# The Spatial-Temporal Transition and Influencing Factors of Green and Low-Carbon Utilization Efficiency of Urban Land in China under the Goal of Carbon Neutralization

**DOI:** 10.3390/ijerph192316149

**Published:** 2022-12-02

**Authors:** Jun Fu, Rui Ding, Yilin Zhang, Tao Zhou, Yiming Du, Yuqi Zhu, Linyu Du, Lina Peng, Jian Zou, Wenqian Xiao

**Affiliations:** 1 College of Big Data Application and Economics (Guiyang College of Big Data Finance), Guizhou University of Finance and Economics, Guiyang 550025, China; 2 Key Laboratory of Green Fintech, Guizhou University of Finance and Economics, Guiyang 550025, China; 3 Key Laboratory Big Data of Statistical Analysis of Guizhou Province, Guiyang 550025, China

**Keywords:** carbon neutrality, GLUEUL, spatial-temporal transition, influencing factors

## Abstract

Urban-land development and utilization is one of the main sources of carbon emissions. Improving the green and low-carbon utilization efficiency of urban land (GLUEUL) under the goal of carbon neutrality is crucial to the low-carbon transition and green development of China’s economy. Combining the concept of green and low-carbon development in urban land use, carbon emissions and industrial-pollution emissions are incorporated into the unexpected outputs of the GLUEUL evaluation system. The super-efficient slacks-based measure (SBM) model, Exploratory Spatial-Temporal Data Analysis (ESTDA) method and Geographically and Temporally Weighted Regression (GTWR) model were used to analyze the spatial-temporal transition and the influencing factors of GLUEUL in 282 cities in China from 2005 to 2020. The result shows that: (1) From 2005 to 2020, the green and low-carbon land-utilization efficiency of Chinese cities shows an increasing temporal-evolution trend, but the gap between cities is gradually widening. (2) From the spatial-temporal dynamic characteristics of Local Indicators of Spatial Association (LISA), regions with the highest GLUEUL have strong dynamics and instability, while cities at the lowest level have a relatively stable spatial structure. On the whole, the local-spatial-transfer direction of GLUEUL of each city is stable, with certain path-dependent characteristics. (3) There are differences in the degree of influence and direction of action of different factors on GLUEUL. The economic development level, industrial-structure upgrading, financial support, wealth level, and green-technology-innovation ability have positive effects on overall GLUEUL, with industrial-structure upgrading promoting GLUEUL the most, while urban population size, foreign-investment scale, and financial-development level play a negative role. This study can provide some empirical and theoretical references for the improvement of GLUEUL.

## 1. Introduction

Urban-land resources are the spatial carrier of social and economic development, and its effective use plays an important role in promoting economic development [1,2]. The development and use of the land during urbanization are considered to be the main source of carbon emissions [3,4]. For a long time, the crude land-use pattern, which emphasizes scale and speed, has consumed a large amount of fossil energy [5]. This has led to a continuous increase in carbon emissions and a continuous deterioration of the social- ecological environment [6,7,8]. It is in stark contrast to the concept of both green and low-carbon development which the core is low energy consumption and low pollutant emissions. However, their emphasis is different. Green development focuses on the impact of pollutant emissions on the ecological environment, while low-carbon development focuses on the impact of carbon emissions on the climate in the process of economic development. Although the concepts of green and low-carbon are slightly different, their directions are very much the same. Implementing the concept of green and low-carbon development into the process of urban-land use and realizing green and low-carbon urban land use has become the key breakthrough for China’s urban- land use.

Due to the scarcity of urban land, the sprawl of construction land, and the rapid growth of urban population, the imbalance between urban-land supply and demand has intensified, and its utilization efficiency has a direct impact on the social and economic development and the construction of the human living environment. Green and low-carbon utilization efficiency of urban land (GLUEUL) is an extension of traditional-land-utilization efficiency, and green low-carbon development is an inevitable choice for China’s development and an important path to break through the developmental bottleneck; it is also becoming a banner leading the general economic and social development in the new era. Under the guidance of green and low-carbon development, the green and low-carbon land use emphasizes moderate use of land resources. While economic output and income are important assessment indicators for the effectiveness of land use, the maintenance of a harmonious relationship between people and land requires urban- land use to take into account related factors, such as the non-degradation of environmental quality, the stability of the ecosystem and the sustainable use of resources, to avoid the inefficient use of land resources. In general, green and low-carbon urban land use is a utilization model that takes into account economic output, resource conservation, environmental protection, and ecological sustainability, and the essence is to integrate the concept of green and low-carbon development into the land-use process to achieve unified economic, social, and ecological benefits of land use.

Under the condition of limited total-urban-land area, actively developing inefficient land, transforming the way of land usage, and promoting intensive, green, and low-carbon land use are key tasks to promote China’s regional sustainable development and ecological-civilization construction. As the world’s largest emitter of CO_2_ [9], China’s carbon emissions and reduction initiatives have been attracting international attention. In response to global climate change, General Secretary Xi Jinping made a solemn pledge to the world at the 75th UN General Assembly General Debate that China strives to achieve the strategic goals of carbon peaking by 2030 and carbon neutrality by 2060. This means that achieving peak carbon and carbon neutrality will be a key element of China’s economic and social development in the medium to long term. Under the constraint of carbon neutrality, introducing the concept of green and low-carbon development into the urban- land-use system, and comprehensively examining the spatial-temporal transition characteristics of GLUEUL and its influencing factors, are essential for achieving the multiple goals of sustainable urban-land use, urban environmental improvement, and high-quality green urban development in China.

The rest of the paper is organized as follows: Section 2 reviews and compares the relevant literature on GLUEUL, Section 3 introduces the data and research methods selected for this paper. Then, Section 4 presents the spatial-temporal transition characteristics of GLUEUL and the spatial-temporal heterogeneity of its influencing factors. Lastly, Section 5 summarizes the findings and gives related countermeasure suggestions.

## 2. Literature Review

At present, academics have explored many issues related to the green and low-carbon use of urban land, mainly focusing on the following two perspectives. First, it is the analysis of urban-land use from a low-carbon perspective [10,11,12]. As the inefficient use of urban land and its disorderly expansion is a typical loss of environmental negative externalities [10]. Some scholars advocate the concept of low carbon as the guide in the process of urban-land use, rational control of resource inputs, and minimization of pollution and carbon emissions, while obtaining high economic, social, and ecological benefits [11]. Its goal is to shift urban-land use from high energy consumption, high pollution with a low-efficiency model, to low energy consumption, low emission with a high-efficiency and high-benefit model [12]. In the evaluation of low-carbon urban-land use, scholars have mainly carried out the empirical analysis of urban-land-use efficiency based on the low-carbon perspective [2,13]. For example, Chen et al. [2] used a non-radial distance function to measure the low-carbon urban-land-use efficiency, and its spatial-temporal transition and influencing factors are combined with kernel-density estimation. Hui et al. [13] used the Durbin model to reveal the relationship between urban form and urban-land use-efficiency in the Yellow River Basin of China under low-carbon constraints. The second perspective is from the green-development concept [1,14,15,16]. Some scholars advocate that the goal of green urban-land use is to achieve a harmonious coexistence between human’s social development and the natural environment in the process of urban-land use [14]. Scholars have also developed related models for measuring urban-land-use efficiency based on the concept of green development and explored the dynamic evolution of urban-land-use efficiency [1,15]. On the basis of these studies, the green-development concept is combined with urban-land-use efficiency, and the driving mechanism of urban-land-use efficiency is systematically analyzed, which further expands the research’s breadth and depth of urban-land use. The existing studies provide theoretical support for guiding the green and low-carbon use of urban land under the goal of carbon neutrality, but there is still room for breakthroughs in terms of research perspectives, measurement methods, and the evolution of spatial and temporal differences.

In terms of research perspectives, urban-land-use studies based on green or low-carbon development concepts have been conducted, mostly around green- development concepts alone or low-carbon constraints. There is a lack of research on the integration of “green” and “low-carbon” into urban-land-use systems, especially on the green and low-carbon use of urban land under the goal of carbon neutrality. In terms of measurement methods, the slacks-based measure (SBM) model [1] usually suffers from the technical problem that multiple decision units are completely valid and cannot be compared separately, while the super-efficient SBM model with unexpected outputs can better solve this problem. In addition to the conventional pollutants [17], the urban-land-use process also emits a large amount of CO_2_. The non-expected output indicators considered in the existing studies cannot fully describe the development level of GLUEUL under the concept of green and low-carbon development; more accurate results can be obtained by incorporating carbon emissions and pollution emissions into unexpected output indicators at the same time. In terms of spatial-temporal variation, methods, such as coefficient of variation [1] and exploratory-spatial data analysis [11], can, to a certain extent, show the characteristics of spatial-temporal variation in urban-land use, but they cannot portray the dynamic evolution of such variation, whereas exploratory spatial-temporal data analysis methods can effectively reveal the dynamic evolution process more comprehensively.

Due to the importance of national policy, China tends to adopt a green and low-carbon perspective on urban-land use. What are the studies being conducted in other parts of the world? Masoudi et al. [18], taking Singapore as an example, study the influence of land use on the composition and cooling capacity of urban green space. Their results show that the land use of urban green space patches with more and simpler shapes, and less fragmentation has a higher cooling effect, which expands the mode of green use of urban land. Becker et al. [19] take the county-level medical units in the United States as an example, studying the relationships between green-land cover and healthcare expenditure. The research shows that more green-land cover helps to improve human health and has a significant negative correlation with healthcare expenditure. Muhamad et al. [20], taking three Southeast Asian cities, Kuala Lumpur, Malaysia, Jakarta, Indonesia, and Manila, Philippines as examples, find that the evolution of green space under rapid expansion can provide support and experience for local urban planning. Since Brazil has one of the greatest biomass potentials in the world, Lap et al. [21] uses Brazil as a case to assess how land-use change affects bioenergy demand, thereby reducing carbon emissions. However, most of the above areas are based on a single green or low-carbon perspective.

In view of this, this paper introduces the concepts of “green” and “low-carbon” development into the urban-land-use system, uses the super-efficient SBM model with unexpected outputs to measure the green and low-carbon land-utilization- efficiency index of China’s urban land under the carbon-neutrality target, and adopts the ESTDA method to reveal the spatial-temporal transition characteristics of China’s GLUEUL. Then, the spatial and temporal heterogeneity of its influencing factors is explored by using a GTWR model, with a view to provide some empirical and theoretical references for urban-land-resource allocation, and achieving green and low-carbon efficient use of urban land.

## 3. Data and Methods

### 3.1. Research Area

**Figure 1:**
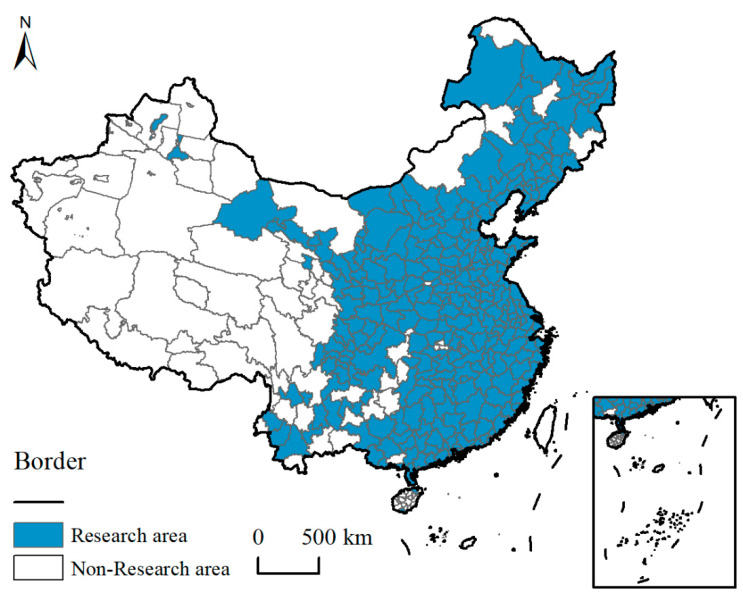
Research area.

Due to the availability and accuracy of the relevant measurement-index data of GLUEUL at the municipal level, cities with missing data are excluded from the study area, and 282 cities in China were finally identified for this study, including Beijing, Tianjin, Shanghai and Chongqing, and 15 vice-provincial cities, including Shenyang, Dalian, Changchun, Harbin, Nanjing, Hangzhou, Ningbo, Xiamen, Jinan, Qingdao, Wuhan, Guangzhou, Shenzhen, Chengdu, and Xi’an, as well as 263 prefecture-level cities, including Shijiazhuang, Tangshan, and Qinhuangdao. The study time points of 2005, 2010, 2015, and 2020, and the specific distribution of the study areas are as follows (Figure 1).

### 3.2. Description of the GLUEUL Measure and Data Sources

Carbon neutrality is China’s emission-reduction target in response to global warming. To achieve the vision of carbon neutrality, it is required to accelerate socio-economic green development and low-carbon transformation. Green and low-carbon development is a strategic choice for China to achieve high-quality and sustainable socio-economic development under severe resource and environmental constraints, in which the concept of green development is an important guiding principle during the period of comprehensive socio-economic transformation, and the concept of low-carbon development is an important guideline for the transformation of production and lifestyle, technological innovation, and energy structure optimization. Specifically for the urban-land- use system, its utilization is regarded as a dynamic process of “input plus output”, and combined with the requirements of carbon-neutral target. The connotation of green and low-carbon use of urban land can be defined as the efficient coupling of “economic, social and environmental” subsystems based on the concept of “green” and “low-carbon” development, and under the multiple constraints of resource input, pollutant emission, and carbon emission. The essence is to maximize social, economic, and ecological output and minimize environmental losses. Concerning the relevant literature (Table 1) and the basic requirements of “reasonable control of inputs, reduction of energy consumption, improvement of green outputs and reduction of pollution emissions” in the process of green and low-carbon use of urban land, an evaluation-index system of GLUEUL is constructed, including three categories of indicators, such as inputs, expected outputs, and unexpected outputs.

In terms of input indicators, this paper selects labor factor, land factor, and capital factor as input indicators. Among them, the total number of urban employments in the year is chosen to characterize the labor factor. Most studies have used the urban-construction-land area to measure the input of the land factor, but the urban-construction-land area is actually the area of land available for use in planning, while the urban-built-up area is the actual area of land for construction, which better reflects the real situation of land input. Therefore, the urban built-up area is chosen to characterize the land factor and the total investment in urban-fixed assets is chosen to characterize the capital factor.

In terms of expected output, this paper selects economic output, social output, and ecological output as indicators. The economic output is characterized by the added value of urban secondary and tertiary industries The social output is characterized by average wages of urban employees. The ecological output is characterized by the total carbon sink of urban green space. The carbon sink of urban green space is mainly the dynamic process of fixing CO_2_ in the air through photosynthesis by urban-green-space vegetation, with the following equation:

(1) $$C_{s}=A_{s}\times f_{s}$$

where, *C_s_* is the total carbon sink of urban green space; *A_s_* is the area of urban green space; *f_s_* is the carbon-sink coefficient of urban green space, referring to the relevant studies on specific carbon-sink coefficients, *f_s_* is taken as 1.66 [27,28].

In terms of unexpected output indicators, this article mainly uses carbon emissions and environmental-pollution emissions in the urban land use process as indicators. Carbon emissions from urban land are measured by carbon emissions from urban construction land, which are indirectly calculated based on the energy consumption of social production and living activities within the urban space. Urban-carbon emissions include carbon emissions from direct energy consumption, such as gas and LPG, as well as carbon emissions from electrical and thermal energy consumption. The carbon emissions from direct energy consumption can be calculated using the relevant conversion factors provided in the IPCC 2006 China Greenhouse Gas Inventory Guidelines. The carbon emissions from electricity consumption are more complex and are based on the approach of Glaeser and Kahn [29]. There is only one emission factor for each regional grid. China’s power grids are divided into six regional grids: north China, northeast China, east China, central China, northwest China, and south China. The baseline emission factors for each regional grid and urban-electricity consumption are used to calculate carbon emissions from electricity consumption in each city. Most of the raw materials used to generate urban heat are raw coal, and the China Urban Construction Statistical Yearbook provides statistics on centralized heat supply in each city over the years. By adding up the carbon emissions from natural gas, LPG, electricity, and heat consumption, the total carbon emissions of each city can be obtained. Environmental-pollution output is measured by industrial emissions, which include industrial wastewater emissions, industrial-sulfur-dioxide emissions, industrial-soot emissions, industrial smoke, dust emissions, and industrial-nitrogen-oxide emissions.

The basic data for the study are obtained from the *China Urban Statistical Yearbook*, the *China Environmental Statistical Yearbook*, and the *China Urban Construction Statistical Yearbook* in previous years, and the missing data are supplemented by the method of substitution of adjacent years or linear interpolation.

### 3.3. Research Methodology

#### 3.3.1. Super-Efficient SBM Models Incorporating Unexpected Outputs

The traditional Charnes-Cooper-Rhodes (CCR) or Banker-Charnes-Cooper (BCC) model is a radial model, that is, the reduction or expansion of input and output must be proportional, which is also the meaning of the word “radial”. Radial data envelopment analysis (DEA) assumes that input and output change in the same proportion. Non-radial DEA relaxes this assumption that input and output can change in different proportions. In the analysis results of this DEA model, there are usually cases in which multiple DMU are evaluated as effective. The maximum efficiency value obtained by the DEA model is 1, and the effective DMU efficiency value is the same. The efficiency of these effective DMUs cannot be further distinguished. In this study, the green low-carbon use efficiency value of urban land calculated by MAXDEA software has a DMU of greater than 1, and the super-efficient SBM model can further distinguish the decision-making units whose efficiency level is greater than or equal to 1, and enhance their comparability. This paper takes 282 cities in China as the research object, each urban-land system is a DMU, and each DMU is composed of input, expected output, and unexpected output. The super-efficient SBM model incorporating unexpected outputs combines the advantages of both the SBM model and the super-efficient DEM model by incorporating unexpected outputs into the model, while allowing for a differentiated comparison of effective decision units, thereby circumventing the problem of information loss from effective decision units, with the following model expression:

(2) $$\rho^{*}=\min\frac{1+\frac{1}{m}\sum_{i=1}^{m}\frac{D_{i}^{-}}{x_{ih}}}{1-\frac{1}{s_{1}+s_{2}}\left(\sum_{r=1}^{s_{1}}\frac{D_{r}^{g}}{y_{rh}^{g}}+\sum_{k=1}^{s_{2}}\frac{D_{k}^{b}}{y_{kh}^{b}}\right)}$$

(3) $$s.t.\{\begin{array}{c} x_{ik}\geq \overset{n}{\underset{j=1,j\neq h}{\sum }}\lambda_{j}x_{ij}-D_{i}^{-},\;i=1,\ldots ,m\\ y_{rh}^{g}\geq \overset{n}{\underset{j=1,j\neq h}{\sum }}\lambda_{j}y_{rj}^{g}+D_{r}^{g},\;r=1,\ldots ,s_{1}\\ y_{kh}^{b}\geq \overset{n}{\underset{j=1,j\neq h}{\sum }}\lambda_{j}y_{kj}^{b}-D_{k}^{b},\;k=1,\ldots ,s_{2}\\ 1-\frac{1}{s_{1}+s_{2}}\left(\overset{s_{1}}{\underset{r=1}{\sum }}\frac{D_{r}^{g}}{y_{rh}^{g}}+\overset{s_{2}}{\underset{k=1}{\sum }}\frac{D_{k}^{b}}{y_{kh}^{b}}\right)>0\\ D^{-}\geq 0,D^{g}\geq 0,D^{b}\geq 0 \end{array}$$

In Formula (2) and (3), the $\rho^{*}$ is GLUEUL, *n* is the number of DMU, *m* is the number of input factors, where *s*_1_ denotes expected output, *s*_2_ denotes unexpected output, and vectors $x\epsilon R^{m},y^{g}\epsilon R^{s_{1}},y^{b}\epsilon R^{s_{2}}$ are the input, expected output, and unexpected output vectors, respectively; matrix $X=\left[x_{1},\ldots ,x_{n}\right]\epsilon R^{m\times n},Y^{g}=\left[y_{1}^{g},\ldots ,y_{n}^{g}\right]\epsilon R^{s_{1}\times n},Y^{b}=\left[y_{1}^{b},\ldots ,y_{n}^{b}\right]\epsilon R^{s_{2}\times n}$. $D^{-}$, $D^{g}$ and $D^{b}$ are the input, expected output, and unexpected output slack variables, respectively, and λ are the weight vectors. In this paper, the efficiency value is calculated by using the MAXDEA software, and the weight *λ* is calculated internally by the software.

#### 3.3.2. Exploratory Spatial-Temporal Data Analysis (ESTDA)

Rey et al. [30,31] proposed that exploratory spatial-temporal data analysis (ESTDA) in-corporates the temporal dimension into exploratory-spatial data analysis, which better compensates the measurement deficiency of ESDA in the temporal dimension. At present, ESTDA mainly includes LISA time path, LISA spatial-temporal transition, and the model has been used in the study of spatial-temporal dynamics analysis of geographic factors with desirable results [32,33,34,35], but the research analysis in the field of spatial-temporal in-teraction characteristics of county-economic resilience is not yet available.

① Global spatial autocorrelation

Testing for the presence of spatial agglomeration in the study area is generally done using global autocorrelation analysis, measured by the Moran index, with the following formula [36].

(4) $$\;I=\frac{n\sum_{a=1}^{n}\sum_{b=1}^{n}W_{ab}\left(X_{a}-\bar{X}\right)\left(X_{b}-\bar{X}\right)}{\left(\sum_{a=1}^{n}\sum_{b=1}^{n}W_{ab}\right)\sum_{a=1}^{n}{\left(X_{a}-\bar{X}\right)}^{2}}$$

where *W_ab_* is the spatial-weight matrix, *X_a_* and *X_b_* are the observations of *a* and *b*, respectively; *n* is the number of spatial cells. If Moran is *I* > 0, it means that there is a positive spatial autocorrelation among the attributes, and the attributes tend to be spatially clustered; conversely, it means that there is a negative spatial autocorrelation among the attributes, and the variables tend to be scattered. If Moran is *I* = 0, it means that the attributes are randomly distributed in space. The closer its absolute value magnitude is to 1, the greater the difference in spatial distribution is represented.

② LISA time path

The LISA time path incorporates a temporal dimension, compared to the traditional static LISA, and is made more dynamic by the transfer characteristics of the LISA coordinates in the Moran scatter plot [37]. ArcGis is a seamless and expanded GIS product family developed by Esri Company in the United States, which is based on industrial standards. The spatial-temporal-synergistic-change characteristics of GLUEUL of each region at the regional scale are visualized in the ArcGIS10.7 software to reflect local spatial differences and spatial-temporal dynamics of GLUEUL changes. In this paper, the relative length, degree of curvature, and direction of movement in the LISA time path index are used for analysis. The relative length reflects the characteristics of local spatial dynamics of regional GLUEUL, the degree of curvature reflects the volatility of the local spatial structure of regional GLUEUL, and the direction of movement reflects the integrative characteristics of the evolution of the local-spatial structure of GLUEUL, and the corresponding formulae are as follows:

(5) $$\;d_{i}=\frac{N\sum_{t=1}^{T-1}d\left(L_{i,\;t},\;L_{i,\;t+1}\right)}{\sum_{i=1}^{N}\sum_{t=1}^{T-1}d\left(L_{i,\;t},\;L_{i,\;t+1}\right)}$$

(6) $$\;\varepsilon_{i}=\frac{\sum_{t=1}^{T-1}d\left(L_{i,t},\;L_{i,t+1}\right)}{d\left(L_{i,t},\;L_{i,T}\right)}$$

(7) $$\;\theta_{i}=\arctan\frac{\sum_{j}sin\theta_{j}}{\sum_{j}cos\theta_{j}}$$

where, $d_{i}$ is the relative length of cell i, and $\varepsilon_{i}$ is the curvature of cell *i*; *N* is the number of study cells; *T* is the length of study time. $L_{i,\;t}$ is the LISA coordinate of cell i at time t. $d\left(L_{i,\;t},\;L_{i,\;t+1}\right)$ is the moving distance of cell i from time *t* to *t* + 1. $d_{i}>1$ represents the mean value of the moving distance of cell i greater than the moving distance. When $d_{i}$ has the larger value, it indicates more dynamic-local-spatial dependencies and spatial structure. For $\varepsilon_{i}$, the larger it is, more dynamic-zigzag-local-spatial-dependence direction and more volatile growth process of the region are indicated.

Here, $\theta_{i}$ is the direction of movement of cell *i*. 0°~90° direction indicates a win-win trend, i.e., the GLUEUL of this cell and the neighboring cells show a high growth trend (relative to the average level, the same below). The 90°~180° direction indicates a lose-win trend, i.e., the GLUEUL of this cell shows a low growth trend, while the neighboring cells show a high growth trend; 180°~270° direction indicates a lose-lose trend, i.e., the GLUEUL of this cell and the directions from 270°~360° indicate a win-lose trend, i.e., the unit has a high growth trend, while the neighboring units have a low growth trend. 0°~90° indicates positive synergistic growth and 180°~270° indicates negative synergistic growth, which indicates that the unit and neighboring units exhibit integrated spatial dynamics.

③ LISA spatial-temporal transition

Ye et al. [38] propose the use of local Markov chains and spatial-temporal transition by em-bedding properties, such as distance, direction, and coalescence of each spatial cell in, Mo-ran’s I scatter diagram at a specific time interval in the traditional Markov chain based on LISA. The spatial-temporal transition is mainly divided into four types *Type*_1_, *Type*_2_, *Type*_3_, and *Type*_4_ (Table 2). *Type*_1_ indicates that it is self-changing and the neighboring units remain unchanged; *Type*_2_ indicates that it remains unchanged and the neighboring units transition; *Type*_3_ indicates that both its own unit and the neighboring units transition; *Type*_3_ can be divided into two types of transition: if the transition direction is the same, *Type*_3*A*_; if the transition direction is opposite, it is *Type*_3*B*_. *Type*_4_ means that neither itself nor its neighboring units transition; Rey defines spatial-temporal variation (SF) and coalescence (*SC*) to represent the spatial-pattern-path dependence and stability characteristics of the study unit, and the ratio of the number of a certain transition type to the total number of transition (*M*) in a certain time period is studied. The formula is as follows:

(8) $$\;SF=\frac{Type_{1}+Type_{2}}{M}$$

(9) $$\;SC=\frac{Type_{3A}+Type_{4}}{M}$$

where, *Type*_1_, *Type*_2_, *Type*_3A_, and *Type*_4_ denote the number of transitions of the corresponding transition type, respectively.

#### 3.3.3. Geographically and Temporally Weighted Regression (GTWR)

In studies related to the spatial differentiation of geographic elements or certain attributes, the advantage of Geographically Weighted Regression (GWR) is that it can reveal the variability of different spatial locations of attributes, and therefore, has a wider range of applications. The disadvantage is that the GWR model does not take the time factor into account, making the model estimates lack explanatory power, while the GTWR model can more effectively deal with spatial-temporal. Its basic equation is as follows:

(10) $$Y_{i}=\beta_{0}\left(\mu_{i},\;v_{i},\;t_{i}\right)+\underset{k}{\sum }\beta_{k}\left(\mu_{i},\;v_{i},\;t_{i}\right)X_{it}+\varepsilon_{i}$$

where, $Y_{i}$ denotes the value of the explanatory variable for the *i*-th sample, and $\left(\mu_{i},\;v_{i},\;t_{i}\right)$ denotes the spatial-temporal coordinates of the *i*-th sample point; $\mu_{i}$ is the longitude of the *i*-th sample point, the $v_{i}$ is the dimension of the *i*-th sample point, and $t_{i}$ is the time of the *i*-th sample point; $\beta_{0}\left(\mu_{i},v_{i},t_{i}\right)$ is the regression coefficient of the *i*-th sample point; $X_{it}$ is the value of the *k*-th independent variable at the *i*-th point; $\varepsilon_{i}$ is the residual; $\beta_{k}\left(\mu_{i},\;v_{i},\;t_{i}\right)$ is the *k*-th regression parameter at the *i*-th sample point. Since the choice of bandwidth affects the establishment of spatial-temporal weights, this paper adopts the AICc law with an adaptive bandwidth.

## 4. The Spatial-Temporal Evolutionary Characteristics of GLUEUL

To reveal the spatial and temporal evolution of GLUEUL 2005–2020, the efficiency values are classified into five levels with the help of the natural-interruption-pointmethod using the ArcGIS 10.7 software: 0.0304~0.1982 is the lowest level, 0.1983~0.2943 is the low level, 0.2944~0.4641 is the medium level, 0.4642~0.7873 is the high level, and 0.7874~1.4590 is the highest level.

### 4.1. Analysis of Temporal Evolutionary Characteristics

**Figure 2:**
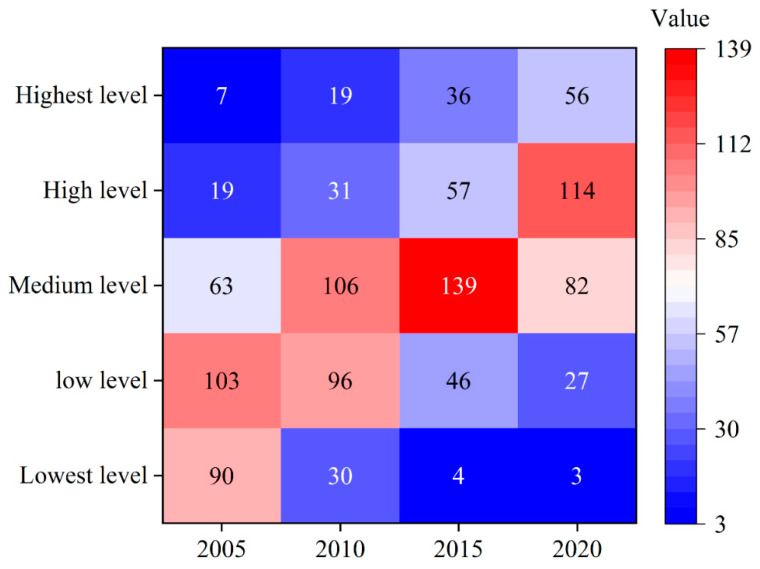
The time-series changes in the number of areas at different levels of GLUEUL from 2005 to 2020.

Figure 2 shows the time-series changes in a number of areas at different levels of GLUEUL from 2005 to 2020. In terms of quantity changes, the number of areas at the lowest level and low level of GLUEUL during the study period shows a linear downward trend, while the number of areas above the medium level shows a significant upward trend, with the number of areas at the high level and the highest level increasing by transitions and bounds from 19 and 7 in 2005 to 114 and 56 in 2020, respectively. In terms of the mean and extreme difference values of GLUEUL, the mean GLUEUL value in 2005 is 0.2828 and the extreme difference value is 1.1664, while the mean efficiency value in 2020 is 0.5787 and the extreme difference value is 1.3415, which indicates that China’s GLUEUL as a whole shows a trend of continuous improvement in time-series evolution, but the gap between cities is getting wider.

### 4.2. Analysis of Spatial Evolutionary Characteristics

**Figure 3:**
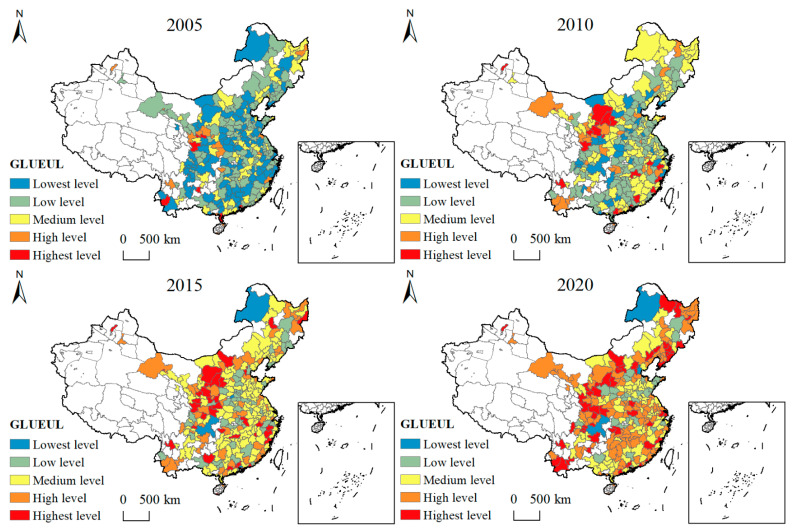
The characteristics of the spatial pattern of GLUEUL from 2005 to 2020.

Figure 3 shows the characteristics of the spatial pattern of urban green and low-carbon land use efficiency from 2005 to 2020. In 2005, the regions with the highest GLUEUL were seven cities, namely Shenzhen, Zhanjiang, Dongguan, Anshun, Lincang, Pingliang, and Longnan, all with the GLUEUL above 1, indicating that these seven cities had a high level of low-carbon green land use, while 90 cities had the lowest GLUEUL and 106 cities had a low GLUEUL. It accounts for 68.4397% of the total, and the overall GLUEUL in China is dominated by the lowest level and low level. In 2020, the number of cities at the highest level increased by 20, reaching 56 cities, mainly located in the Heihe and Pu’er linkage zone, and the number of cities at the high level also increased to 114. The total number of high level and highest level cities is 60.2837%. Overall, the level of green and low-carbon land use in China’s cities is on an increasing trend.

### 4.3. Analysis of Spatial-Temporal Transition and Influencing Factors of GLUEUL

#### 4.3.1. Spatial Autocorrelation Features

Using the ArcGIS10.7 software to conduct global-autocorrelation analysis, the global Moran index of 282 cities in China was obtained (Table 3). The global Moran’s I and Z values of GLUEUL passed the 1% test under four-time sections, and the Moran’s I values were all greater than 0, with the global Moran’s I and Z values from 2005 to 2020 showing a slow increase, indicating that the spatial agglomeration effect of GLUEUL was increasing, and the spatial divergence characteristics of GLUEUL were not entirely determined by random factors, but the spatial-spillover effect plays a key role in its formation.

#### 4.3.2. Spatial Characteristics of the LISA Time Path

**Figure 4:**
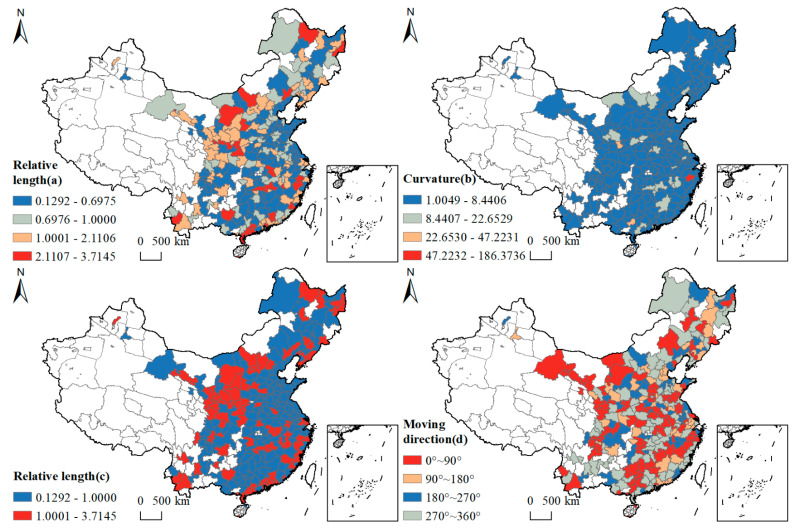
Spatial distribution of LISA time path characteristics of GLUEUL, 2005–2020.

The relative lengths reflect the local spatial dynamics of the area. The relative lengths of the GLUEUL of 282 cities were visualized in ArcGIS 10.7, and the relative length values were divided into four classes using the natural-breakpoint method with interrupted values of 0.6975, 1.0000, 2.1106, and 3.7145, respectively. As shown in Figure 4a, there were 101 cities with relative length values greater than 1 during the study period, accounting for 35.8156% of the study area, and the GLUEUL of these cities had a more dynamic local-spatial structure. In terms of spatial distribution, as shown in Figure 4c, cities with relative lengths greater than 1 were mainly concentrated on the western side of the “Hu Huan Yong” line and the southeastern coastal region, with a small number of scattered distributions in the central region. There were 181 cities with a relative length of less than 1, accounting for 64.1844% of the study area, mainly clustered in the eastern and central regions of China.

The curvature reflects the local-spatial-dependence direction of the dynamic curvature of the region. The curvature of GLUEUL of 282 cities is visualized in ArcGIS 10.7, and it is divided into four classes with interruption values of 8.4406, 22.6529, 47.2231, and 186.3736, using the natural-interruption-point method. As shown in Figure 4b, the curvature values in the study area are all greater than 1, and the spatial-temporal interaction between cities plays an important role in the dynamic evolution of GLUEUL, reflecting the existence of dynamic changes in green low-carbon land use between cities and their neighboring cities. These cities with a large curvature are Jinhua, Xianyang, and Guigang, which have obvious instability in the direction of spatial transfer, while the rest of the cities have a lower curvature and relatively stable spatial-transfer direction. The mean value of curvature is 4.5337, and there are 228 cities below the mean value, accounting for 80.8511%, indicating that on the one hand, the GLUEUL of most cities has a relatively independent-development path and is less influenced by the surrounding cities; on the other hand, the interaction between cities with high GLUEUL and their neighboring cities with low efficiency still has much room for improvement, and the role of radiation needs to be further enhanced.

The movement direction reflects the different growth trends of regions and neighboring regions, visualizing the movement direction of 282 cities in ArcGIS 10.7. As shown in Figure 4d, there are 144 regions with synergistic growth in the movement direction of the LISA time path of GLUEUL, accounting for 51.0638% of the study area, indicating that GLUEUL has good integration conditions and has a basis for synergistic development. These 99 cities with positive synergistic growth, accounting for 35.1064%, show the characteristics of synergistic high-speed growth of GLUEUL with obvious block-like agglomeration characteristics, and 45 cities with negative synergistic growth, accounting for 15.9574% of the study area, show the characteristics of synergistic low-speed growth with obvious fragmented distribution characteristics.

#### 4.3.3. Spatial Characteristics of the LISA Spatial-Temporal Transition

Using GLUEUL’s Moran’s *I* scatter plot coordinates, the spatial-temporal transition probabilities are calculated for the period of 2005–2020. As can be seen from Table 4, the most common type of transition is *Type*_4_. Moran’s *I* is always located in the same quadrant, which means that neither itself nor its neighboring areas are transitioning, and the probability of this type of occurring is 57.57%, indicating that the GLUEUL in most cities remains relatively stable from 2005 to 2020, with no significant state shifts occurring. The next type is *Type*_1_, which indicates a transition of its own without a transition in its neighborhood, with a probability of 20.33% for *Type*_1_. Then, it is *Type*_2_, which means that there is no transition in itself but a transition in the adjacent area; the probability of occurrence of *Type*_2_ is 16.67%. The probability of occurrence of Type_3_ is 5.44%, which indicates that both they and their neighbors have transitioned, and the possibility of double transition transfer is very small, indicating that the local spatial structure of GLUEUL is relatively stable. The spatial-temporal cohesiveness of GLUEUL is 61.11%; the spatial-transfer variation is not significant, and there is a certain “path dependence” phenomenon.

### 4.4. Analysis of Factors Influencing GLUEUL

The spatial-temporal transition in China’s GLUEUL is the result of the combined effect of many factors. Based on comprehensive and scientific principles, this paper explores the factors influencing the spatial-temporal transition of China’s GLUEUL under the goal of carbon neutralization from eight aspects: economic development level (EDL), industrial structure upgrading (ISU), urban population size (UPS), financial support (FS), foreign investment scale (FIS), financial development level (FDL), wealth level (WL) and green technology innovation capability (GTIA). Cities with high EDL generally have higher green and low-carbon land use efficiency, which is measured by per capita GDP. The secondary industry is generally characterized by high energy consumption and high emissions. The more reasonable the industrial structure is, the higher the industrial efficiency per unit land area is, which is more conducive to the improvement of GLUEUL. The tertiary sector as a proportion of the secondary sector is applied to measure the ISU. As an important driving force of urban development under the concept of green development, the urban population is often accompanied by large-scale development and utilization of urban land in the process of concentration in urban areas. The concentration of population will promote the accumulation of resources, generate economies of scale and improve the efficiency of green land use, but it may also increase the cost of congestion and environmental pressure, and thus, inhibiting the improvement of GLUEUL. Therefore, the number of people in urban areas of the city is selected to measure UPS. Local governments tend to expand the size of cities and increase the intensity of urban-land development. Accumulating original capital through land finance, attracting enterprise investment, and improving the efficiency of unit-land output in these areas are conducive to the improvement of GLUEUL. Nevertheless, it may also attract a large number of low-quality enterprises in the process of “land investment”, resulting in a large number of industrial-pollution emissions, further causing the reduction of GLUEUL. FS is represented by the local-general-budget expenditure as a proportion of GDP. On the one hand, the investment of foreign enterprises may bring advanced green and low-carbon technologies to the local; on the other hand, it can also bring high-pollution and high-emission industries, and thus affecting GLUEUL. Here, we use the total output value of foreign-invested enterprises as a proportion of GDP to measure the FIS. Then, FDL may help enterprises to invest in projects with high emission and high pollution, which is not conducive to the improvement of GLUEUL. It chooses the year-end loan balance as a percentage of GDP to measure. The higher the WL, the more people’s consumption concept will improve, and they are more willing to consume high-end green products, forcing enterprises to carry out green transformation. The average wage of urban workers is selected to measure the WL. GTIA helps to improve the green low-carbon technology of enterprises, and it is measured by the number of green-patent applications, as shown in Table 5.

#### 4.4.1. Data Testing

In this paper, GTWR regression models were conducted to test the factors influencing GLUEUL at four time cross-sections. The relevant parameters of GTWR are shown in Table 6. In terms of goodness of fit, R^2^ and adjusted R^2^ are higher than 0.79, and the model is well-fitted.

#### 4.4.2. Spatial and Temporal Variation Characteristics of Influencing Factors

Figure 5, Figure 6, Figure 7, Figure 8, Figure 9, Figure 10, Figure 11 and Figure 12 show that there are spatial and temporal differences in the direction and intensity of the influence of the individual influences on GLUEUL over the four time periods 2005–2020, specifically.

**Figure 5:**
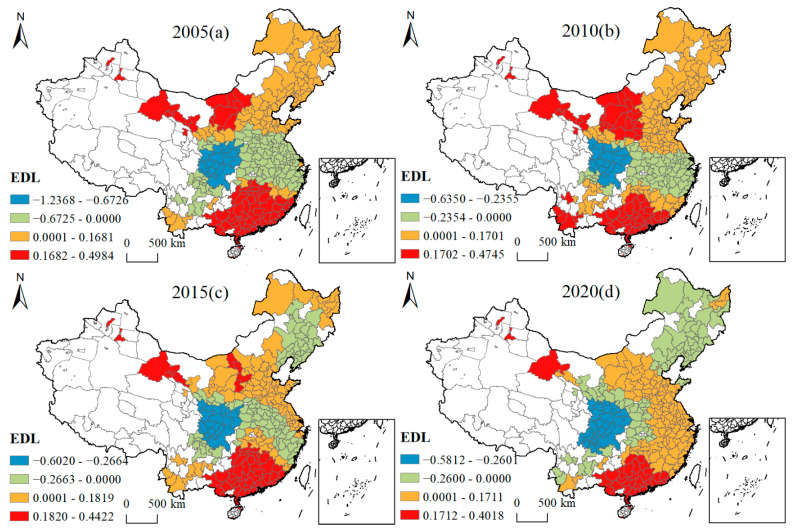
Four-year regression coefficients of EDL.

The average influence of EDL is positive, with the average influence increasing and the spatial-temporal non-stationarity weakening. As can be seen from Figure 5, in terms of the spatial-temporal divergence of EDL’s influence, in 2005, EDL was mainly a facilitating influence in southern and northern China, and a negative influence in central China, where EDL had the strongest positive influence in central Inner Mongolia, northwestern Gansu Province, Urumqi and Karamay in Xinjiang, Guangxi Province, Guangdong Province, Fujian Province, and some cities in Jiangxi Province. In 2020, Guangdong Province, Guangxi Province, Jiuquan City in Gansu Province, Urumqi, and Karamay City in Xinjiang were still among the highest positive-impact regions, and the regions with the strongest negative impacts were Chengdu, Chongqing, southeastern Gansu, and southern Shaanxi cities. The average regression coefficient of EDL increased from 0.0027 to 0.0107, as China clearly put forward the “double carbon” strategic goal in 2020; under the carbon neutrality target, the green and low-carbon development of the economy was maintained, which promoted the improvement of GLUEUL. The extreme difference of the regression coefficient decreased from 1.7351 to 0.9830, and the regional imbalance in the degree of influence of EDL on GLUEUL decreased over time.

**Figure 6:**
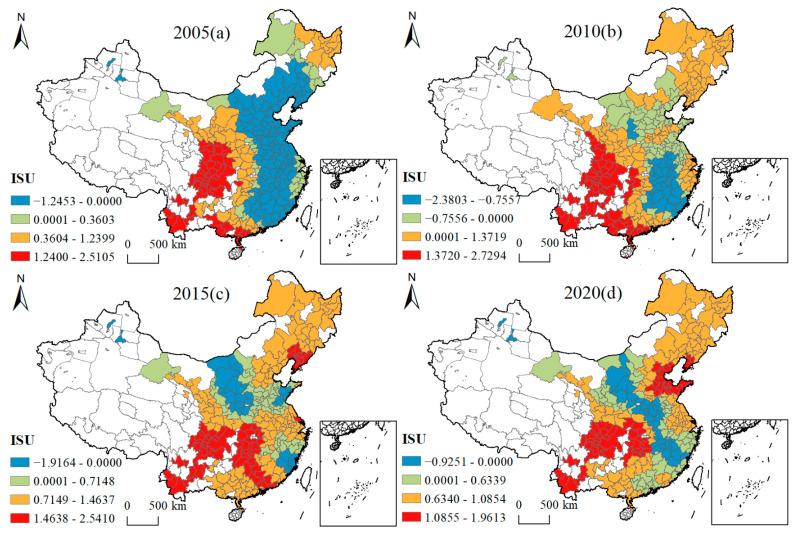
Four-year regression coefficients of ISU.

The average influence of ISU is positive, with an increase in average influence and a weakening of spatial-temporal non-stationarity. As can be seen from Figure 6, in terms of the spatial and temporal divergence of ISU’s influence, in 2005, it had a positive influence on cities in western China and a negative influence on cities in the east, and having the strongest positive influence on Sichuan Province, Chongqing Municipality, southeast Gansu, southwest Shaanxi, Yunnan Province, Guangxi, and southern Guangdong. In 2020, the influence of ISU had obvious characteristics of spatial differentiation. ISU had an inhibitory effect on the cities on the axis of Ordos and Sanming, and had a positive impact on other areas. The area with a high degree of positive influence was transferred from some cities in Gansu, Shaanxi, Guangxi, and Guangdong, to Hunan and Hubei. The average regression coefficient of ISU increased from 0.3092 to 0.6944, which shows that ISU had a positive effect on the improvement of GLUEUL in most cities, while cities with mainly secondary industries tended to emit more pollutants per unit of land, and thus, having a negative effect on GLUEUL. The extreme difference in regression coefficients decreased from 3.7557 to 2.8864, and although the spatial variability of ISU’s impact on GLUEUL decreased over time, the regional imbalance was still large.

**Figure 7:**
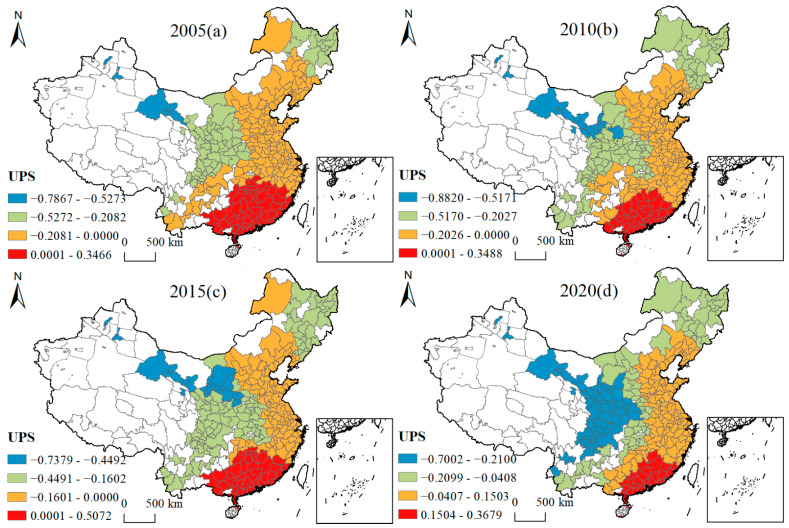
Four-year regression coefficients of UPS.

The average influence of UPS is negative, with the negative average influence decreasing and the spatial-temporal non-stationarity slightly weakening. As can be seen from Figure 7, in terms of the spatial-temporal divergence of UPS influence, in 2005, UPS had a negative influence on most cities in China, with the highest negative influence in Zhangye, Jiuquan, Urumqi, and Karamay, and positively influenced areas concentrated in Guangxi Province, Guangdong Province, Hunan Province, Jiangxi Province, and Fujian Province. In 2020, the negatively influenced areas expanded to Shaanxi Province, Sichuan Province, Chongqing City, and some cities in Yunnan and Hebei. While Shandong, Jiangsu, Anhui, and Zhejiang in the eastern region turned from negative influence to positive influence. The average regression coefficient for UPS changed from −0.097 to −0.0386, indicating that the concentration of urban population increased the cost of congestion and environmental pressure, and had a negative impact on GLUEUL. The extreme difference in regression coefficients decreased from 1.1332 to 1.0681, with a slight reduction in the regional imbalance to the extent of the impact of UPS on GLUEUL over time.

**Figure 8:**
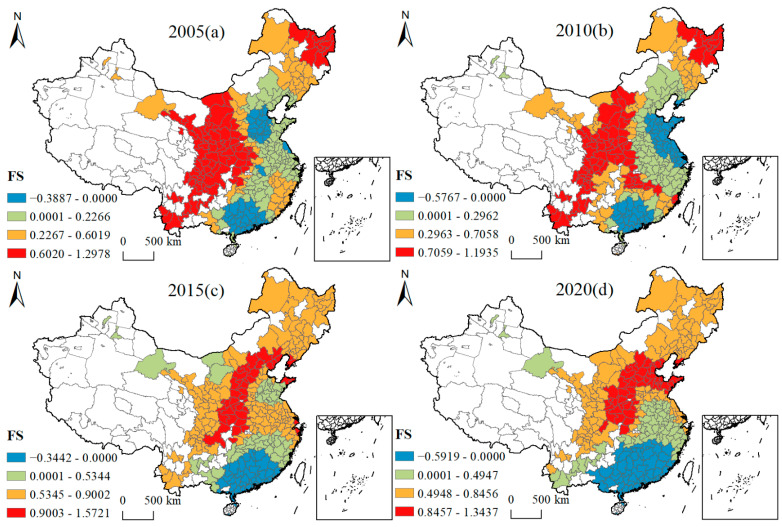
Four-year regression coefficients of FS.

The average influence of FS is positive, with increased average influence and spatial-temporal non-stationarity. As can be seen from Figure 8, in terms of the spatial-temporal divergence of the influence of FS, in 2005, the majority of Chinese cities were positively influenced by FS, with the regions with the highest positive influence being Gansu, Yunnan, Guiyang, Sichuan, Chongqing, Shaanxi, Henan, and Heilongjiang provinces, and the regions with a negative influence concentrated in some cities in Guangxi, Guangdong, Hebei, and Shandong provinces. In 2020, the regions with the highest degree of positive influence shifted to cities in the Beijing-Tianjin-Hebei urban agglomeration, Shaanxi, Shandong, western Henan, and western Hubei, and the negatively influenced regions shifted to Jiangxi, Fujian, Hunan, Guangdong, and Guangxi provinces. The average regression coefficient of FS increased from 0.3629 to 0.4413, and the government’s accumulation of primary capital through land finance played an important role in improving urban infrastructure and attracting investment, which improved the efficiency of land output per unit in these regions, and thus, increased GLUEUL. The extreme difference of the regression coefficient increased from 1.6865 to 1.9356, and the regional imbalance in the degree of FS’ impact on GLUEUL had been widening over time.

**Figure 9:**
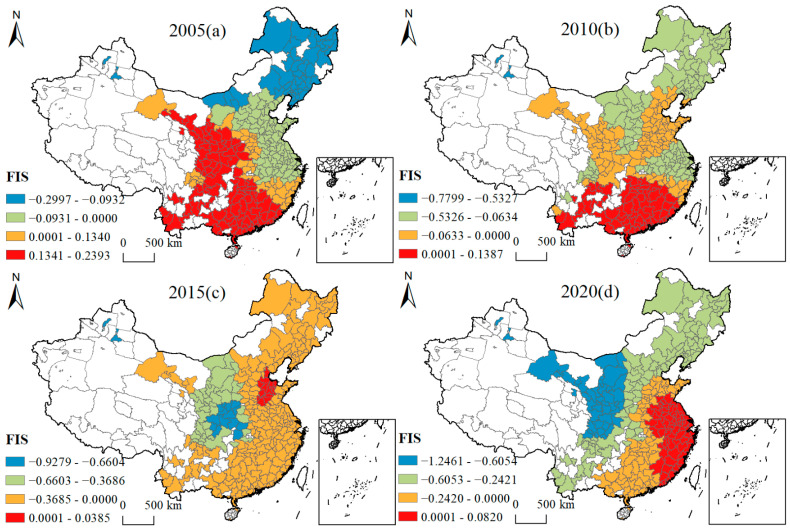
Four-year regression coefficients of FIS.

The average influence of FIS shifted from positive to negative influence, and the spatial-temporal non-stationarity increased. As can be seen from Figure 9, from the spatial-temporal divergence of FIS influence, in 2005, the direction of FIS influence on Chinese cities gradually changed from negative to positive influence from northeast to southwest. The early northeast region was dominated by heavy industries, and foreign investment brought highly polluting and high-emission industries to the region, resulting in a negative influence on GLUEUL. In 2020, the influence of FIS on Chinese cities had gradually changed from negative to positive from the northwest to the southeast. The reason for this may be that foreign-invested enterprises brought more advanced green and low-carbon technologies to the southeast coastal region, which contributed to the improvement of GLUEUL. The average regression coefficient of FIS decreased from 0.0512 to −0.2539, indicating that foreign-invested enterprises caused high pollution emissions in most cities, resulting in a negative impact on GLUEUL. The extreme difference of the regression coefficient increased from 0.5391 to 1.3281, and the regional imbalance in the degree of FIS’ impact on GLUEUL had been widening over time.

The average influence of FDL is negative, with the average negative influence rising and the spatial and temporal non-stationarity weakening. In 2005, FDL had a negative impact on cities in northern and central China, with the strongest negative impact in Heilongjiang, Jilin, Liaoning, Shaanxi, Jiangsu, Anhui, and Zhejiang provinces, and a positive impact on cities in southern China. In 2020, the negative impact area shifted to the line between Gansu Province and Fujian Province, and the positive impact area shifted to Liaoning Province, Hebei Province, Shandong Province, Beijing City, and Tianjin City. The average regression coefficient of FDL shifted from −0.0113 to −0.0262. Overall, the increase in FDL helped enterprises to invest in projects with high pollution and emissions, leading to an increase in carbon emissions and environmental-pollutant emissions, which in turn, had a negative impact on GLUEUL, with the extreme difference of the regression coefficient decreasing from 0.2496 to 0.1780. The regional imbalance in the extent of FDL’s impact on GLUEUL was narrowing over time.

**Figure 11:**
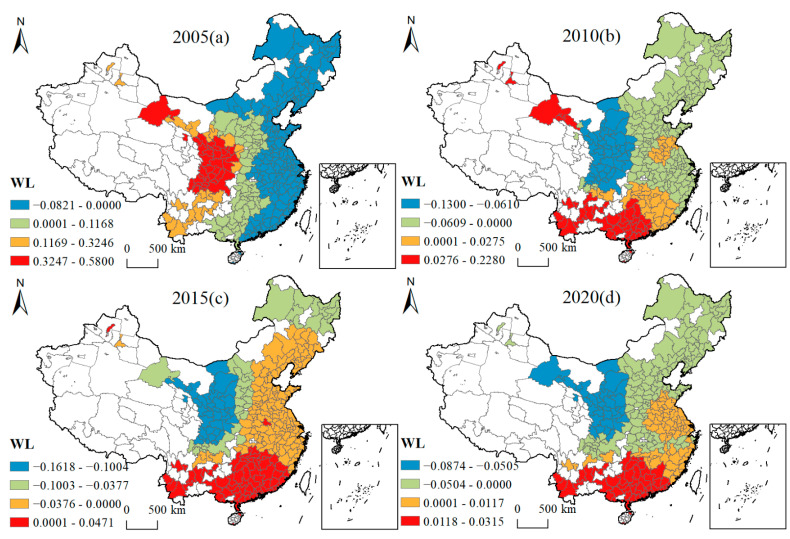
Four-year regression coefficients of WL.

The average influence of WL is positive, the average influence decreases and the spatial-temporal non-stationarity diminishes. As can be seen from Figure 11, in terms of the spatial-temporal divergence of WL’s influence, the direction of WL’s influence on Chinese cities gradually changed from negative to positive from east to west in 2005. In 2020, the direction of WL’s influence on Chinese cities gradually changed from negative to positive from the north to the south. The average regression coefficient of WL decreased from 0.0689 to 0.0113. The positive influence of WL on GLUEUL decreased, probably due to the lack of awareness of people’s purchase of green products and the lack of motivation of enterprises to innovate and transform green products. The regression coefficient extreme difference value decreased from 0.6621 to 0.1189, and the regional imbalance in the degree of WL’s influence on GLUEUL was decreasing over time.

**Figure 12:**
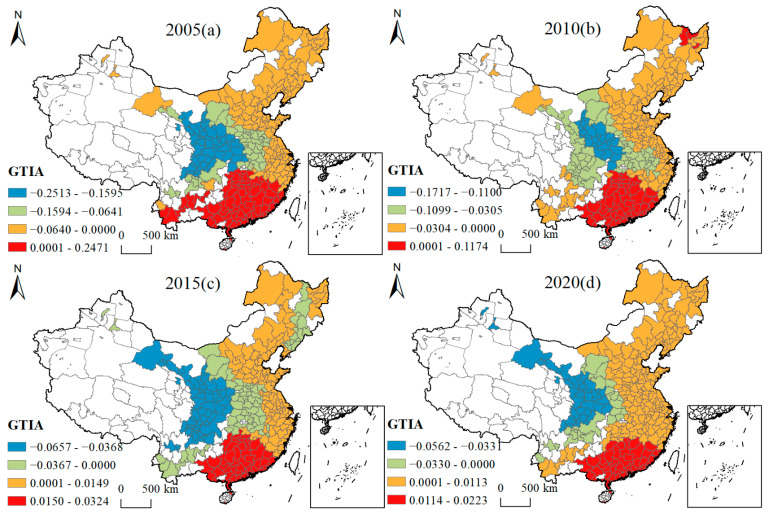
Four-year regression coefficients of GTIA.

The average influence of GTIA is positive, with the average influence rising and the spatial-temporal non-stationarity weakening. As can be seen from Figure 12, from the spatial-temporal divergence of GTIA’s influence, in 2005, GTIA had a positive influence on Hunan Province, Jiangxi Province, Fujian Province, Guangxi, Zhuang Autonomous Region, and Guangdong Province, and a negative influence on the other regions. In 2020, the positive influence region had shrunk, with cities in Hunan Province, Jiangxi Province, and northern Fujian Province turning from positive to negative influence, with the strongest degree of negative influence concentrated in Gansu, Sichuan, and Shaanxi Provinces. The average regression coefficient of GTIA increased from 0.0007 to 0.0284, indicating that green-technology development contributed to the improvement of green low-carbon technology and GLUEUL. The extreme difference value decreased from 0.4984 to 0.0786, and the regional imbalance in the degree of GTIA’s impact on GLUEUL was narrowing significantly over time.

## 5. Conclusions

### 5.1. Main Conclusions

Based on the 282 cities in China, this paper uses the super-efficient SBM model to measure GLUEUL by using carbon emissions and environmental-pollution emissions as unexpected outputs. Then, the ESTDA-GTWR framework is conducted to explore the spatial and temporal transition characteristics of GLUEUL and its influencing factors, with the following main findings.

(1) During the study period, the average GLUEUL value in China increased from 0.2828 in 2005 to 0.5787 in 2020, with GLUEUL as a whole showed an increasing trend. In terms of spatial evolution, the number of areas with high and highest efficiency values increased from 19 and 7, respectively, in 2005 to 114 and 56, respectively, in 2020, and the extreme difference in GLUEUL expanding from 1.1664 to 1.3415, with its dynamic evolution process featuring obvious regional differences and gradually increasing disparities between cities.

(2) In terms of the LISA time-paths study, the areas with high GLUEUL are strongly dynamic and unstable, mainly on the western side of the “Hu Huan Yong” line and the southeastern coastal region, while the areas with low GLUEUL have a relatively stable local spatial structure, mainly in the eastern and central regions of China. The lower GLUEUL areas have a relatively stable local spatial structure, which is mainly in eastern and central China. From the spatial characteristics of the LISA spatial and temporal transitions of each city, most cities in China do not experience significant spatial and temporal transitions, and the local spatial structure of each city’s GLUEUL and neighboring cities is relatively stable, with a high degree of spatial cohesion and a certain “path locking” characteristic.

(3) There are differences in the degree and influential direction of EDL, ISU, UPS, FS, FIS, FDL, WL, and Getia on GLUEUL. EDL, ISU, FS, WL, and Getia have a positive influence on China’s overall GLUEUL, with ISU having the greatest promoting effect. Highly polluting industrial enterprises cause large amounts of CO_2_ emissions, so the more rational the industrial structure, the higher the industrial efficiency per unit land area, the more conducive it is to the improvement of GLUEUL, followed by FS. The financial support from the local government is conducive to the coordination of land resources and the improvement of land-resource-allocation efficiency. UPS, FIS, and FDL play a negative role on China’s overall GLUEUL.

### 5.2. Recommendations

Combining the spatial and temporal transition characteristics of China’s GLUEUL and its influencing factors, under the constraints of the carbon-neutrality target, the path of improving China’s GLUEUL can be explored through measures such as accelerating the high-quality transformation of industries, improving the financial-support system, and promoting green-technology innovation and application.

(1) Accelerate the high-quality transformation of industries. On the one hand, with the help of ISU, the urban-land-use structure will be optimized, and urban-land-use indicators will be tilted towards low energy consumption, low pollution, and high-efficiency industries, to reduce the unexpected output of urban-land use and enhance economic output. On the other hand, through the green transformation of industries, backward-production capacity will be eliminated, new production capacity will be generated, and the mechanism of energy-saving-and-consumption reduction in the whole process of industries will be improved. The spatial-reconfiguration effect of industries will be used to reduce the scale of regional pollution emissions and promote the development of urban-land use in the direction of green and low-carbon.

(2) Improve the financial support system: Accelerate the reform of the land market, establish a sound land-market ecology, explore the construction of a diversified land supply mechanism, tap into the potential of idle and inefficient land use, and improve the efficiency of land resource allocation are some of the ways forward; other suggestions include: accelerate the implementation of special policies on financial support for green and low-carbon development, use carbon-emission-reduction support tools, and provide sufficient financial resources for the development of green and low-carbon industries; additional recommendations are: promote the construction of a carbon-emission trading market and give full play to its incentive effect on green and low-carbon land use.

(3) Promote green-technology innovation and application. Advantages of an intellectually and technologically dense city to promote the emergence of green and low-carbon technologies and their transfer and diffusion to other low-tech areas can be leveraged. Research and development of low-carbon frontier technologies, such as carbon capture, sequestration, and utilization, can be strengthened to seize the peak of green low-carbon technologies. Pollution reduction, energy saving, and renewable-energy technologies to urban land use activities can be applied; carbon emissions and pollution emissions in the process of urban land use can be reduced, and sustainable use of urban land resources can be promoted. The establishment of standards in the field of green low-carbon technologies and the certification system of the origin of low-carbon products can be promoted, and high-tech innovative talents can be actively introduced to ensure the long-term and stable development of green-technology innovations.

## Figures and Tables

**Figure 10:**
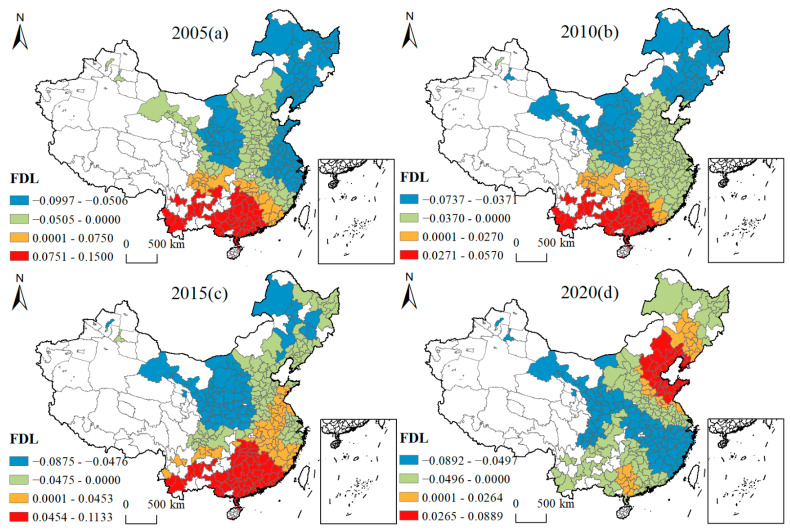
Four-year regression coefficients of FDL.

**Table 1:** Input and output indicators of GLUEUL.

Index	Specific Index	Indicator Description	References
Input	Labor input	Total number of urban employments in the year (unit: 10,000 people)	Han et al. [17] Wang et al. [22] Xu et al. [23]
	Land input	Urban built-up area (unit: km^2^)	Koroso et al. [24]
	Capital input	Total investment in urban fixed assets (unit: 100 million yuan)	Han et al. [17]
Expected output	Economic output	Added value of urban secondary and tertiary industries (unit: 10,000 yuan)	Wang et al. [22] Xu et al. [23]
	Social output	Average wages of urban employees (unit: yuan)	Xie et al. [25]
	Ecological output	Total carbon sink of urban green space (unit: 10,000 tons)	Tan et al. [1]
Unexpected output	Carbon-emission output	Carbon emissions of urban construction land (unit: 10,000 tons)	Shan et al. [26]
	Environmental-pollution output	Industrial pollution emissions (unit: 10,000 tons)	Han et al. [17]

**Table 2:** Basic types of spatial-temporal transition.

Type	Spatial-Temporal Transition Type	Symbolic Expressions
*Type*_1_ type	Self-transition, neighborhood stabilization	HH_t_→LH_t+1_, LH_t_→HH_t+1_, HL_t_→LL_t+1_, LL_t_→HL_t+1_
*Type*_2_ type	Self-stabilization, neighborhood transition	HH_t_→HL_t+1_, LH_t_→LL_t+1_, HL_t_→HH_t+1_, LL_t_→LH_t+1_
*Type*_3_ type	Self and neighbor transition	HH_t_→LL_t+1_, LL_t_→HH_t+1_, LH_t_→HL_t+1_, HL_t_→LH_t+1_
*Type*_4_ type	Self and neighboring stabilization	HH_t_→HH_t+1_, HL_t_→HL_t+1_, LL_t_→LL_t+1_, LH_t_→LH_t+1_

**Table 3:** Four-year global autocorrelation Moran’s *I* and Z values for GLUEUL in China.

Year	Moran’s *I*	Z-Value	*p*-Value
2005	0.0748	3.8459	0.0001
2010	0.0878	4.4491	0.0000
2015	0.0951	4.7729	0.0000
2020	0.1305	6.4725	0.0000

**Table 4:** Spatial-temporal transition matrix for Moran scatter plot of GLUEUL.

t/t + 1	HH	LH	LL	HL	Type	Quantity	Percentage	SF	SC
HH	0.1442	0.0355	0.0165	0.0496	*Type*_1_	172	0.2033	0.3700	0.6111
LH	0.0414	0.0827	0.0366	0.0083	*Type*_2_	141	0.1667		
LL	0.0189	0.0343	0.1217	0.0733	*Type*_3_	46	0.0544		
HL	0.0461	0.0106	0.0532	0.2270	*Type*_4_	487	0.5757		

**Table 5:** Influencing factors of GLUEUL.

Influencing Factors	Description	References
EDL	GDP per capita (yuan)	Gao et al. [39]
ISU	Tertiary sector as a proportion of secondary sector (%)	Chen et al. [40] Xu et al. [41]
UPS	Number of people in urban areas of the city (10,000)	Liu et al. [42] Li et al. [43]
FS	Local-general-budget expenditure as a proportion of GDP (%)	Tu et al. [44]
FIS	Total output value of foreign-invested enterprises as a proportion of GDP (%)	Luo et al. [45]
FDL	Year-end loan balance as a percentage of GDP (%)	Li et al. [46] Hussain et al. [47]
WL	Average wage of urban workers (yuan)	Wen et al. [48]
GTIA	Number of green patent applications (pcs)	He et al. [49]

**Table 6:** Parameters of relevance for GTWR.

Model Parameters	Sigma	Residual Squares	AICc	R^2^	Adjusted R^2^	Spatial-Temporal Distance Ratio
Value	0.1851	38.6297	−602	0.7978	0.7934	0.1

## Data Availability

The data presented in this study are available on request from the corresponding author.
